# The National Heatwave Plan – A Brief Evaluation of Issues for Frontline Health Staff

**DOI:** 10.1371/currents.dis.aa63b5ff4cdaf47f1dc6bf44921afe93

**Published:** 2014-01-13

**Authors:** Chris Boyson, Sarah Taylor, Lisa Page

**Affiliations:** Wye Valley NHS Trust, Hereford, Herefordshire, United Kingdom; Brighton and Sussex Medical School, United Kingdom; Harrogate District Foundation Trust, United Kingdom; Sussex Partnership NHS Foundation Trust, Brighton, United Kingdom; Brighton & Sussex Medical School, United Kingdom

## Abstract

Background: The adverse effects of heatwaves on mortality are well recognised. Heatwaves are predicted to become more frequent and severe in coming decades. England’s National Heatwave Plan (NHP) aims to prepare the country for periods of extreme heat and thereby limit adverse health effects. The central aim of this study is to understand how effectively the NHP is disseminated within an acute hospital and to identify any barriers to its use. Methods: Qualitative data was collected through semi-structured interviews and focus groups with key hospital managers, nurses and healthcare assistants. All participants were recruited from a single hospital in the South East of England. Data were analysed using Framework Analysis. Results: We conducted two focus groups with frontline clinical staff and five interviews with senior managers, all of whom deemed the NHP a low priority. Hospital managers showed good awareness of the plan, which was lacking amongst frontline staff. Nevertheless front line staff were familiar with the dangers of excess heat and felt that they individualised care accordingly. Communication of information between managers and frontline staff was highlighted as a problem during heatwaves. Additionally, issues with inadequate building stock and equipment limited effective implementation of the plan. Participants were able to suggest novel improvements to the plan. Conclusions: Increased awareness and improved communication could help better integrate the NHP into the clinical practice of English hospital-based healthcare professionals. Further evaluation of the NHP in acute care trusts and other health care settings is warranted to expand upon these initial findings.

## Introduction

The adverse health effects of heatwaves were seen across Europe in 2003, when 14,729 excess deaths occurred in France during a 20-day heatwave - equivalent to a 55% rise in mortality on baseline periods.^1^ Although temperatures in England were not as extreme, 2,091 excess deaths were still reported.^2^Following the 2003 heatwave, the first public health contingency plan for England was introduced entitled the ‘National Heatwave Plan’ (NHP).^3^ Since 2004 there have been several updates to the English NHP and now similar policies are in effect in many other countries, including France.

The NHP aims to increase awareness of the threat of heatwaves amongst health and social care services as well as the general public. By improving preparedness, the expectation is that adverse health impacts of heatwaves will be reduced. The NHP outlines short and long term actions for the preparation of, and reaction to a heatwave. **Table 1 **describes some of these strategies specific to a hospital environment.^3^

**Table 1 - Examples of short and long term heat-negating strategies in a hospital environment.** Edited from Department of Health, 2011^3^

Installation of thermometers in patient bays and recording of temperature four times daily
Identifying vulnerable individuals
Creation of cool areas for high risk patients
Obtain supplies of ice and cold water
Turning off unnecessary lights and electrical equipment
Maximising green areas on and around the hospital site
New building designs to aid passive cooling and minimise carbon emissions

The NHP is based on a heat-health watch system that describes four levels of caution (see **Figure 1**).^3^ These levels are dependent on regional pre-defined temperatures. Level 1 is activated during the summer months and becomes the baseline for that period. Each level guides the implementation of a range of actions by health and social care sectors.

**The National Heatwave Plan’s heat-health watch system:**
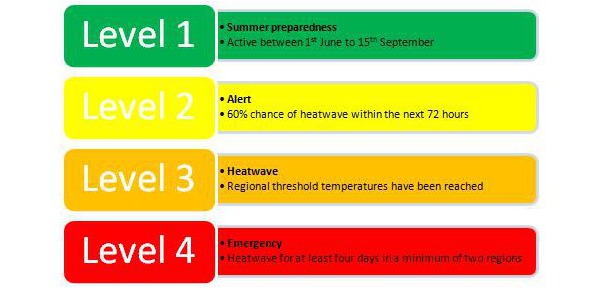

There is international evidence to suggest that the introduction of heatwave response plans may limit excess mortality rates during times of excess heat.^4^^,^ 5 ^,^ 6 A French study suggested that 4,388 excess deaths were prevented during a 2006 heatwave after the introduction of the country’s heatwave plan two years previously.^6^^,^ 7

The UK Health Protection Agency (now known as Public Health England) has produced three commentaries on the English NHP since its inception.The first and most comprehensive of these was produced in 2007 and concluded that most healthcare managers were aware of the plan and found it helpful for heatwave planning and response.^8^ The 2008 and 2011 reports were largely confined to seminar discussions, held prior to each update of the NHP.^9^^,^ 10 The 2008 commentary reviewed the temperature thresholds and terminology used in the heat-health watch system and explored methods of cooling buildings and urban environments.^11^ The 2011 seminar focused on the comparison of the NHP with the recently published National Cold Weather Plan, the use of early mortality and morbidity data and a drive to produce effective public information messages regarding heatwaves.^10^^,^ 11

Despite these commentaries, little empirical evaluation of the English NHP has been undertaken**. **One qualitative study looked at the feasibility and perceptions of the plan amongst frontline health, social and voluntary staff in a community setting.^12^ The study found that most participants were unaware of the document and perceived heatwaves as a low threat.^12^ However, 56% of deaths attributed to the 2003 heatwave in England took place in hospitals, a figure which cannot be fully explained by the increase in hospital admissions.^13^ This suggests that hospitals may in themselves be risky environments for vulnerable patients,^14^ and yet the effectiveness of the NHP within hospitals has never been evaluated.

The objectives of this study are to:

i.) assess how effectively the English NHP is disseminated amongst hospital staff; and

ii.) identify any factors that may inhibit the NHP’s implementation.

## Methods

Before beginning research ethics approval was sought and granted from Brighton and Sussex Medical School Research Governance and Ethics Committee.

This study was performed using a qualitative analysis. Using a qualitative method allowed us to explore the motivations and behaviours related to the implementation of the NHD.

Data was collected in two forms; focus groups comprised of nurses and healthcare assistants (HCAs) (i.e. frontline staff) and semi-structured interviews with hospital managers from the same hospital. The rationale behind selecting managers and frontline hospital staff was due to a concern raised in previous reports about poor communication between the two groups.^8^^,^ 9 ^,^ 12 We wanted to be able to explore how top-down communication strategies affected dissemination of the NHP.

**Focus groups**

Eligibility for focus groups included nurses and HCAs who had held their current role in the chosen hospital since at least the previous summer (defined as 1^st^June 2011). Temporary staff were excluded. Nurses and HCA's were the main participants, and mixed within the focus groups, as they are likely to take the lead on implementing and monitoring many of the ward based actions recommended in the NHP. Potential participants were recruited using a variety of methods, as described in **Figure 2**, who subsequently formed two focus groups.

**Focus Group Recruitment:**
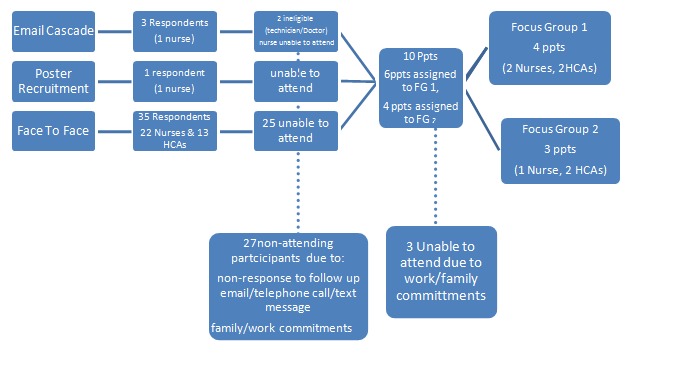
The focus group recruitment process, reasons for non-participation and end-result focus groups participant numbers. FG = focus group Ppt = participant HCA = health care assistant

**Semi-structured interviews**

Hospital managers of interest were identified using the trust’s staff directory and approached via email. Managers whose roles were most directly relevant to the implementation of the NHP were selected. Again, participants must have held their current role since at least the previous summer. Five interviews took place in participants’ offices and lasted between 30-45 minutes each. The decision was made not to include hospital managers in the focus groups as it may have affected the dynamics of the focus groups. However the researchers did understand that semi-structured interviews are susceptible to bias as participants may feel the need to present their own work in a positive light.

**Preparation for data collection**

Topics for both focus groups and interviews were developed by the principal researchers. Questions were developed and debated by the research team after consulting the NHP and relevant literature. The researchers also sought the views of relevant professionals to ensure relevance of the topics posed; these included a Health Protection Agency regional director and original lead author of the NHP, a health scientist from the Met Office and hospital redevelopment architects. Agreed topics included how widely the heatwave plan was circulated and acknowledged, the value given to the document, areas of the plan that are or are not implemented and the reasons behind these, and considerations for future editions of the plan. Although most questions were common to both interviewees and focus group participants, questions were customised accordingly. Topic guides for both the semi-structure interviews and the focus groups have been included below in figure 3 and figure 4.

**Focus Group Topic Guide:**
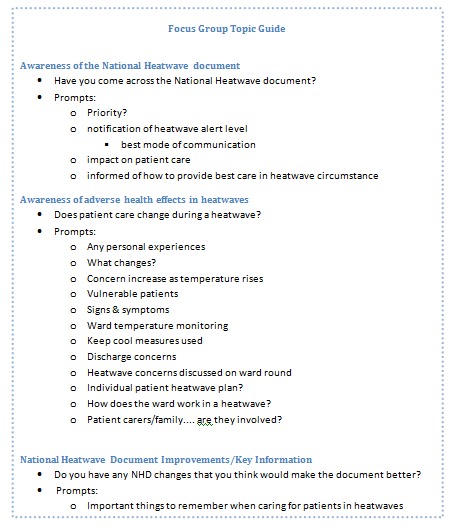

**Semi-structured interview topic guide:**
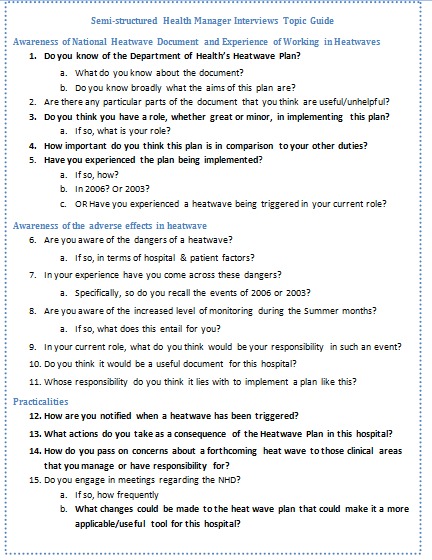

**Data collection**

The study was carried out at a large acute care hospital in England. The hospital covers a catchment area of around 300,000 people and offers a diverse range of healthcare services. Recruitment and data collection occurred in January and February 2012. All participants were given information sheets and provided with an opportunity to have any queries answered by the researchers. Written informed consent was obtained to audio record the interviews and focus groups. Care was taken not to disclose specifics of the project to the participants prior to data collection; instead, the participants were informed that the discussion would be about how hospitals cope during certain weather conditions.

It is important to recognize that data collection occurred in a non-heatwave period. The last Level 2 (alert) status in the area had been issued in 2011 and the last Level 3 (heatwave) status was triggered in 2009. Level 4 has never been activated.

**Data Analysis**

All transcripts were anonymised and transcribed verbatim. A Framework Analysis was used to analyse focus groups transcripts, this meant that no prior assumptions were made about the knowledge or opinions that would emerge from these groups. Framework analysis is less appropriate for semi-structured interviews, especially given the ‘expert’ role held by the hospital managers. Instead, themes identified in the focus groups were mapped back onto interview transcripts in order to compare and contrast opinions.

Two researchers independently immersed themselves in the transcripts and noted emergent concepts, from which a preliminary list of themes were developed. These initial themes were discussed with a third researcher to arbitrate data that was more challenging to classify. At this point any data which directly challenged the emerging themes was discussed with the independent researcher and reasons for these differences were sought. Categories were then derived for each theme. Once no more themes emerged from the focus group data, the themes and categories were mapped back onto the interview transcripts. This mapping process was done by reading the interview texts, whilst continually referring to the focus group themes and categories and noting where there were commonalities and differences. Relevant quotes were coded and charted in Microsoft Excel until all relevant data had been scrutinised and the classification was agreed upon by all researchers.

## Results

**Focus groups**

Using a Framework Analysis approach, four themes emerged from the focus group discussions with frontline staff. These can be summarised as follows:

1) barriers to implementation,

2) importance of communication,

3) natural knowledge of heat and health; and

4) suggested improvements.

The themes are expanded upon below and the categories that emerged for each theme described below. There are examples of data extracted for each theme in each section.

**1) Barriers to implementation**

The extent to which the NHP was implemented by staff in the observed hospital appeared variable. The following factors may have contributed to this limitation:

*Awareness*

Most frontline staff were unaware of the NHP or the local trust version of the document. Only one participant claimed to have known about the document. This difference of awareness between participants was discussed with the independent researcher and it was thought this could be due to some participants having more recent training/induction into the trust where information on the NHP can be found.

"No... I’ve not seen or heard anything about it."

*Priority*

There was a frequently stated view that the risk of heatwaves was too small to warrant the use of heatwave strategies when compared to other daily duties. Many participants also remarked on there being a greater cause for concern during extreme winter weather events, such as snow.

"As far as heatwaves are concerned it will probably be quite low down on my list of priorities."

*Lack of suitable resources*

Many concerns were raised regarding limited or unsuitable resources and facilities, which may limit the heat-negating measures that can be employed. This ranged from a lack of functioning equipment, such as ice machines and fans, to unsuitable outdated hospital buildings. Frontline staff also identified poorly regulated heating controls on wards; whether due to unsuitable building stock or lack of communication. Many respondents pointed to the contribution of the hospital building itself to adverse heat effects. One respondent appreciated the need for taking heatwaves into account when designing new builds.

"If they only have ‘x’ number of air conditioning units to go around a whole building you are not necessarily going to get it."

*Conflicting actions and a lack of solutions*

Frontline staff noted that some actions recommended in the NHP would conflict with other guidelines. Examples included the beneficial effects of using fans against the potential infection control risk and the extent to which windows were allowed to be opened. Participants perceived that there was little or no guidance on how these conflicts should be resolved.

**2) Importance of communication**

Frontline staff felt that communication was vital in achieving heatwave preparedness. The following categories were identified within this theme:

*Manner of communication*

Despite email being the hospital’s preferred method of communication for disseminating information in the event of a heatwave, some participants felt that email was a poor method of communication; the remaining respondents were unclear how an alert should be communicated.

"If you look at the pick up rate of any email you probably wouldn’t bother sending it."

*Ongoing communication*

Some frontline staff suggested that there was a lack of two-way communication during heatwave periods. There was also a consensus that HCA and nursing concerns were often overlooked by medical or managerial staff during a heatwave.

"If someone was to do something about it, it [recording temperatures] wouldn't be pointless."

***3) Natural knowledge of heat and health***

Despite frontline staff being unaware of the NHP, respondents demonstrated reasonable knowledge of how heat and health are linked and appropriate actions that should be taken in order to optimise care:

*Identifying vulnerability*

Participants were able to identify many of the at-risk groups identified within the NHP, particularly the elderly and those with certain medical conditions.

*Heat-negating actions*

Even though participants were largely unaware of the NHP, they could successfully outline simple actions that they would undertake during periods of excess heat, many of which overlap with NHP guidance.

"It's just vigilance... shutting the blinds, opening windows... taking blankets away... encouraging fluids."

*Common sense but worthwhile*

Many heat-negating actions were deemed to be ‘common sense’ by participants. Despite this, participants still felt the idea of a NHP was worthwhile. They felt it could be a useful document for reference and optimising patient care. A couple of participants also pointed out that the NHP may eventually impact on the country’s societal perception of heatwaves.

*Clinical experience and individualised care*

Frontline staff noted the importance of clinical experience during heatwaves, however there was a consensus that changes to individual patient care would only ever be subtle. Respondents rejected the idea that mass care should change when a heatwave occurs and instead the view that healthcare professionals should still manage patients on an individual level was voiced.

"I think it [patient care in heatwaves] becomes more intuitive the longer you have been working on a ward."

*Importance of patients’ discharge*

The NHP discusses the extra difficulties that arise when planning patients’ discharge during a heatwave period. Participants seemed attentive to this risk.

***4) Suggested improvements***

Frontline staff were keen to volunteer ideas that may improve future versions of the NHP, these include:

*Greater *use of media**

Although the NHP states that media use should be encouraged if a Level 2 or higher alert is triggered, none of the respondents could recall seeing or hearing a public media message regarding heatwaves. The use of television advertisements were suggested.

"...maybe television adverts when the weathers got the potential to be hotter... to make people more aware."

*Family involvement*

Respondents felt family involvement was important in protecting those susceptible to excessive heat. They suggested that public media messages could be targeted towards relatives of those who may be isolated or those who may struggle to look after themselves.

*Care of staff*

Frontline staff mentioned that they themselves found it more difficult working during hot conditions and that this should be taken into consideration in future iterations of the plan.

"It’s just suffocating [heat] I don’t know how we carry on with it!"

**Interviews**

The themes and categories described above were used to probe the managers’ interview transcripts. The similarities and differences between frontline staff and managers’ perspectives are highlighted below.

**i) Similarities**

*Low priority*

Like frontline staff, hospital managers deemed the threat of heatwaves to be small and thus the aims and actions of the NHP were considered a low priority.

"We luckily don’t see too many high temperatures so it’s one of the lower risk ones..."

*Lack of suitable resources*

Managers largely agreed with frontline staff that lack of resources and poor building design often limits the benefit of staff actions. However, managers explored this conflict further and attempted to balance perceived constraints against what can be achieved. Managers were hopeful that a proposed redevelopment of the observed hospital would resolve some of these difficulties.

"We actually have to hire in extra air con units and it’s fairly crude, it’s sort of hose through the window..."

*Common sense but worthwhile*

Hospital managers felt that many of the suggestions stated in the NHP were common sense. Despite this, managers usually felt the NHP was worthwhile and they appeared to attach greater importance to it than frontline hospital staff.

"It’s the art of the blindingly obvious isn’t it really which it’s extraordinary to see it written down. But actually that’s important....... then it won’t be taken for granted."

*Identifying vulnerability*

Like frontline staff, managers identified the elderly as being particularly at risk of the adverse effects of excess heat. Several other at-risk patient groups were also recognised .

**ii) Differences**

*Good awareness*

All hospital managers were aware of the NHP compared to just one frontline employee.

*Effective communication*

Methods of communication regarding the presence or prediction of a heatwave appear to have been well thought out at a managerial level. Key managers are informed about heatwave alerts both by email and verbally during daily operational meetings. Managers have also considered the possibility of using instant messaging and social networking sites to disseminate alerts quickly to all staff. The consensus amongst managers was that communication had been well thought through, yet according to frontline focus groups, the cascading of information was ambiguous and incomplete. The resilience manager identified that this may be a problem.

"The text messages the quickest way I get it [heatwave alert], although I will get the formal email as well and I’ll just get another message for each level."

*NHP too comprehensive*

A common criticism of the NHP amongst hospital managers was that some sections were irrelevant to their role. The creation of a local trust version of the policy helped with this issue; however some interviewees suggested that separate documents should be created for hospital and community services.

## Conclusions

**Summary of main findings**

We found that hospital managers showed good awareness and knowledge of the NHP, in contrast, frontline clinical staff were largely unaware of it. Despite this, frontline staff demonstrated reasonable knowledge of the effects of heat on health and felt able to use their clinical experience to identify vulnerable individuals. Several barriers to implementation of the NHP were identified and communication of the plan’s actions to frontline staff is probably not as successful as managers assume. Staff were able to suggest improvements to the NHP.

**Discussion of main findings**

The NHP is circulated amongst senior managers and directors from a variety of organisations including acute hospital trusts, social care services, voluntary organisations and public health bodies. This accounts for the high degree of awareness shown by hospital managers compared with the lack of familiarity amongst frontline ward staff. A previous study has remarked on a similar discrepancy in a community context.^12^ However, participants in our study unanimously believed that heatwaves were a low priority due to their perceived low likelihood – this was the case for managerial staff who were familiar with the NHP as well as frontline staff who were not. The research was carried out in the winter months, so findings may have been different had the study been conducted during heatwave season. This lack of priority given to heatwaves is not unique to hospitals, but has been found amongst healthcare professionals in a community setting and the general public.^12^,^15^

We suggest that the priority given to the NHP within an organization is likely to correlate with the degree to which its actions are implemented and it was clear that both managers and frontline healthcare staff deemed the NHP as a low priority. Further raising of awareness amongst frontline health and social care professionals to the existence and aims of the NHP may be needed before its full implementation is possible.

All staff face the difficulty of implementing the NHP amongst other competing priorities. Added to this, frontline staff highlighted the sub-optimal building environments in which they often work and the difficulties of implementing the NHP recommendations in poorly designed wards, with poorly maintained equipment. We recognise this could be quite an individual issue raised within the hospital where the participants worked. Interestingly participants from different areas of the hospital placed varying amount of importance on their environment depending upon the design of the ward. The hospital studied in this paper has incorporated many of the NHP’s suggested actions into their building redevelopment project.

Communication is important in all contingency planning but is particularly critical during heatwave periods. Quick and effective communication and clearly established procedures are essential since the time lag between the start of a heatwave and excess mortality is brief.^14^,^15^ Communication with the Met Office and intra-managerial communication appeared to be working well; however, in common with other reports, the filtering of messages to frontline staff was poor.^8^,^12^ Although the NHP advises on the importance of communication between staff it does not provide specific guidance on how an alert message, starting at the Met Office, should be cascaded down, level by level to frontline staff. Despite the 2008 HPA report recommending the inclusion of a flowchart to resolve the issue, this has not yet materialised.^8^

The managers that we interviewed were critical that the NHP covers information on many different sectors, resulting in much of the material being viewed as irrelevant by individual establishments. This concern has been partially addressed in the 2013 update of the NHP.^16^ Many acute trusts, including the one in this study, have created their own local version of the plan, which essentially removes material that is not relevant to a hospital environment.^19^,^20^,^21^,^22^ The French heatwave plan has adopted this method and has separate plans for nursing homes, hospitals, voluntary care services and fire and police services.^7^ Such tailored planning may help overcome two difficulties with the current plan; a lack of clear responsibility for who implements the plan and ambiguity regarding how messages should be communicated to frontline clinical staff.

Finally, although the plan does promote the use of public media messages forewarning the arrival of a heatwave, many respondents denied ever seeing or hearing any such advice. This may be because there had been no recent heatwaves prior to our study. In contrast, a previous study found that recall of media coverage was high when participants were questioned just months after a heatwave.^8^ Either way, public health messages about the dangers of heatwaves need to be regularly reinforced to the general public. One possibility is the use of a media campaign just prior to the beginning of summer when people are most susceptible to adverse heat effects. Alternatively warnings could be incorporated during weather forecasts; this would be cheap and achieve high coverage. Relatives of those who may be vulnerable to extreme heat, (e.g. the elderly, socially isolated and very young) also need to be aware of these dangers and to know what they can do to help. ****

**Strengths of the study**

Most focus group participants worked on hospital wards that were located in the oldest part of the hospital and were therefore most affected by hot weather. The hospital is also located in the South East of England, which, other than London, is the region most likely to be exposed to high temperatures. This is useful, as the staff involved in the study are those most likely to be caring for patients during a real heatwave.

Our study participants were all hospital staff, meaning that this is the first report to evaluate the use of the NHP solely in a hospital environment. Furthermore, because we included managerial and clinical staff from the same hospital, the study reveals contrasts between managers’ perceptions and their staff’s knowledge and behaviour.

**Limitations of the study**

We cannot be confident that our focus groups were sufficiently large or numerous to achieve saturation with respect to the themes and categories that emerged during data analysis. A larger number of participants in the focus groups would have allowed us to randomise participants to different focus groups in efforts to aid discussion regarding heatwave issues experienced on a larger number of wards/variety of hospitals. If we had been able to gather data from a variety of hospitals this would have allowed us to triangulate our data adding validity to our results. We had hoped to present the results of the study back to the participants to ensure they were a thorough representation of participant views however due to the time restraints of the study this did not occur.

It is important to note that the study was carried out during a relatively cool period of weather, the previous regional heatwave being in 2009. We can therefore speculate that participant’s responses may have been different had there been a more recent heatwave or had data collection occurred in the summer months. For instance, we postulate that respondent’s perceptions on the priority of heatwave preparation may differ during warmer conditions. Nevertheless this allowed us to explore the longevity of the NHP, something that previous reports could not comment on.

**What this study adds**

This is the second independent evaluation of the use of NHP since its inception in 2004 and is the first to examine in detail the use of the plan in a hospital setting. Our study has begun to reveal different barriers to implementation to those previously identified in a community setting and has identified key areas that, with more research, could be improved upon in future iterations of the NHP.

**Recommendations for future research**

- We have highlighted the need to evaluate the knowledge and behaviours of those who are responsible for implementing the NHP. Ongoing more extensive evaluation of the implementation of the NHP needs to continue in future years.

- Our study demonstrates ongoing problems with top-down communication of the NHP, these problems may be under-estimated by managers and need further exploration. Email appears to be an ineffective form of communication for frontline staff and future studies may consider testing other modes of communication, particularly looking into the value of instant messaging services and social networking websites.

- The use of the NHP in other institutional settings needs to be evaluated. For example, several studies have highlighted the increased risk of those with psychiatric illness during hot weather.^8^,^23^,^24^,^25^ With limited ability to open windows or mobilise patients to cool areas, psychiatric hospitals may pose an especially challenging environment to keep cool and yet use of the NHP in this environment has not been evaluated.

## Competing Interests

The authors declare that no competing interests exist.
